# Evaluating Multimodal Large Language Model (LLM) (Generative Pre-trained Transformer 5 (GPT-5)) for Meniscal Tear Detection on Knee Magnetic Resonance Imaging (MRI): A Pilot Study

**DOI:** 10.7759/cureus.99472

**Published:** 2025-12-17

**Authors:** Kwan Kit Chan, Wai Hoi Chan, Lok Chi Chan

**Affiliations:** 1 Department of Radiology, North District Hospital, Hong Kong, HKG; 2 Department of Orthopedics and Traumatology, Princess Margaret Hospital, Hong Kong, HKG

**Keywords:** ai and machine learning, gpt-5, knee mri, large language model, meniscal tear

## Abstract

Introduction

Magnetic resonance imaging (MRI) of the knee is the gold standard for evaluating meniscal injuries. While specialized artificial intelligence (AI) models have demonstrated high diagnostic capability in detecting meniscal tears, the performance of general-purpose large language models (LLMs) with multimodal vision capabilities remains underexplored. Previous iterations, such as generative pre-trained transformer 4 (GPT-4) (OpenAI, San Francisco, CA, USA) with vision, have shown limited success in direct musculoskeletal image interpretation. This study evaluates the diagnostic performance of the latest-generation LLM, generative pre-trained transformer 5 (GPT-5), in detecting meniscal tears on knee MRI.

Objectives

This study aimed to evaluate the diagnostic performance of GPT-5 (a general-purpose multimodal LLM) in detecting meniscal tears on knee MRI in a zero-shot setting, using a publicly available dataset.

Materials and methods

One hundred knee MRI examinations (50 with meniscal tears, 50 without) were randomly selected from the MRNet validation dataset, with ground-truth labels extracted from the dataset. Sagittal T2-weighted and coronal T1-weighted series were reviewed for completeness and image quality and then converted to Portable Network Graphics (PNG) slices. GPT-5 (gpt-5-2025-08-07) analyzed each case in zero-shot fashion using a fixed prompt requesting a binary ("yes/no") determination of meniscal tear presence without any clinical context. Model predictions were compared with ground truth, and accuracy, precision, recall, specificity, and F1-scores were calculated with 95% confidence intervals.

Results

GPT-5 achieved an overall accuracy of 76% (95% CI: 0.668-0.833). The model demonstrated a sensitivity (recall) of 84% (95% CI: 0.715-0.917) and a specificity of 68% (95% CI: 0.542-0.792). The precision for detecting tears was 72.4%, and the F1-score was 0.778.

Conclusion

In this pilot study, GPT-5 demonstrates potential in the zero-shot interpretation of knee MRIs for meniscal tear detection, outperforming previous multimodal LLMs. However, the results should be interpreted with caution due to study limitations, and clinical utility is currently limited by a high false-positive rate and lack of visual explainability. Nevertheless, this pilot evaluation provides an initial proof of concept, and with larger datasets, rigorous validation, improved calibration, and enhanced explainability, future multimodal LLMs may evolve into supportive, human-in-the-loop tools in musculoskeletal radiology.

## Introduction

Magnetic resonance imaging (MRI) of the knee is the standard for evaluating meniscal injuries, and more knee MRIs are performed than for any other musculoskeletal region [1]. Prior studies report that MRI achieves high sensitivity and specificity for meniscal tears (approximately 83-94% and 82-93%, respectively) when compared against arthroscopic findings [2]. In recent years, artificial intelligence (AI) tools have been explored to assist in image interpretation. Convolutional neural network (CNN) models (e.g., Stanford's MRNet) have demonstrated high diagnostic capability on knee MRI, with an area under the curve (AUC) ~0.85 for meniscal tear detection and accuracy around 80 [3]. Notably, MRNet's performance showed no significant difference from general radiologists on many metrics, aside from radiologists having higher specificity for meniscal tears [3].

Large language models (LLMs) with multimodal vision capability represent a new frontier in medical imaging AI. Early efforts have yielded mixed results. Generative pre-trained transformer 4 (GPT-4) (OpenAI, San Francisco, CA, USA) with vision (GPT-4V) has struggled with direct image-based diagnosis: one study found GPT-4V attained only 8% diagnostic accuracy on musculoskeletal radiology cases, far below a radiology resident (41%) or attending (53%) [4]. In contrast, text-based GPT-4 (without image input) can perform better when provided structured imaging findings [5], but that approach still relies on human-generated descriptions. Generative pre-trained transformer 5 (GPT-5) is the latest-generation LLM purported to have enhanced vision analysis capabilities, with its performance in interpreting medical images remaining unproven. In this study, we evaluate GPT-5's ability to detect meniscal tears on knee MRI, using 100 knee MRI cases from a public dataset (50 with meniscal tears, 50 without meniscal tears).

## Materials and methods

Study design

This retrospective study did not directly involve human subjects. All data were completely devoid of any identifying information and were publicly available; therefore, institutional review board approval was not required.

Objective

This study aimed to evaluate the diagnostic performance of GPT-5 (a general-purpose multimodal LLM) in detecting meniscal tears on knee MRI in a zero-shot setting, using a publicly available dataset.

Dataset

This study utilized knee MRI examinations from the publicly available MRNet dataset (Stanford Machine Learning Group) [6], derived from the study "Deep Learning for Diagnosis of Knee Injuries from MRI: MRNet" [3]. The MRNet dataset comprises 1,370 knee MRI examinations, with each examination containing three MRI series: sagittal T1-weighted, coronal T2-weighted, and axial proton density-weighted sequences. All series were represented as sequences of 2D slices, and each slice consisted of 256×256 pixels. Patient imaging was originally acquired at either 1.5T or 3T magnetic field strength. Each examination received binary labels for abnormality, anterior cruciate ligament (ACL) tear, and meniscal tear, which were derived from clinical radiology reports. To ensure patient privacy protection, the dataset excludes demographic information and individual scanner parameters. There were no retained metadata in filenames or Digital Imaging and Communications in Medicine (DICOM) tags preserved in the dataset.

Within the 1,370 examinations, 120 cases comprised the validation set. Binary labels for this validation subset were established through majority vote consensus among three practicing, board-certified musculoskeletal radiologists from a large academic institution (years in practice: 6-19 years; mean: 12 years). These radiologists had access to all DICOM series, original clinical reports, and follow-up imaging during their interpretation [3]. Individual MRI knee examinations were stored in separate .npy files organized by imaging sequence (axial, sagittal, and coronal).

Image selection and preparation

A total of 100 MRI knee examinations were randomly selected from the validation set, consisting of 50 positive cases (meniscal tear present) and 50 negative cases (no meniscal tear). This 1:1 class ratio was deliberately maintained to ensure the balanced assessment of sensitivity and specificity. Analysis was restricted to sagittal T2-weighted and coronal T1-weighted series.

Inclusion criteria were as follows: (1) complete imaging series in both sagittal and coronal planes encompassing the entire knee joint and (2) adequate image quality without artifacts (e.g., motion-related artifacts). Exclusion criteria included incomplete imaging series, missing ground-truth diagnostic labels, or poor image quality due to artifacts. All images underwent manual visual inspection to verify image quality (confirming no distortion of image from the resized image in the database), consistency, orientation, and proper slice ordering prior to analysis.

Image analysis procedure

For each MRI examination, sagittal and coronal image series were retrieved from the dataset. Pixel intensities were normalized to the 0-1 range using min-max scaling prior to conversion into Portable Network Graphics (PNG) format. All images consisted of 256×256 pixels, with individual series containing between 20 and 44 slices. Images were submitted to GPT-5 (model: gpt-5-2025-08-07) via OpenAI's application programming interface (API). No annotations, markings, or clinical information were provided to the model.

A standardized prompt was designed to elicit a binary response regarding the presence of a meniscal tear in a case-based approach (analyzing the sagittal and coronal image series at the same time): "Analyze these knee MRI images (coronal and sagittal views) and determine whether meniscal tear presents. Respond only with 'yes' or 'no'." GPT-5 returned a single-word response, either "Yes" (tear present) or "No" (no tear), for each case. All responses were recorded. All inferences were conducted in zero-shot mode, with no fine-tuning or task-specific retraining of GPT-5 performed. The model received no additional clinical context or patient information during analysis.

Statistical analysis

Model predictions were compared to the ground-truth labels. We calculated standard classification metrics: accuracy, precision, recall, F1-score, and support. Calculated were 95% confidence intervals (CIs) for the proportion-based metrics (accuracy, precision, recall, specificity, negative predictive value (NPV)) using the Wilson score method. For the F1-score, non-parametric bootstrapping at the case level was used to compute the 95% CI.

## Results

**Figure 1:**
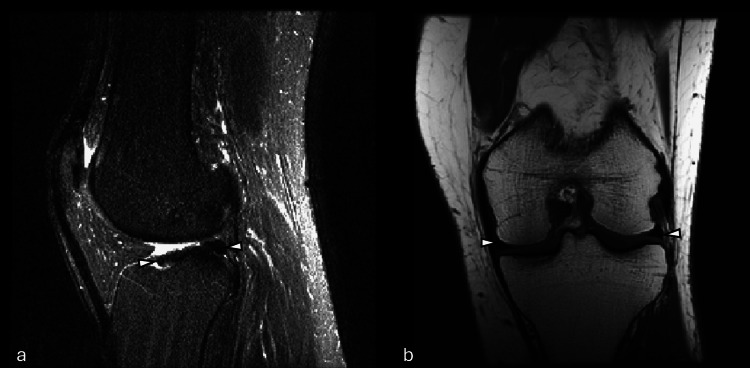
Example of MRI in which GPT-5 incorrectly identified a meniscal tear despite an intact meniscus (false positive). Sample images from (a) sagittal plane T2-weighted series and (b) coronal plane T1-weighted series showing a knee with no meniscal tear (arrowheads). This sample illustrates a false-positive case, as GPT-5 erroneously classified the image as positive for a tear. GPT-5: generative pre-trained transformer 5; MRI: magnetic resonance imaging

**Figure 2:**
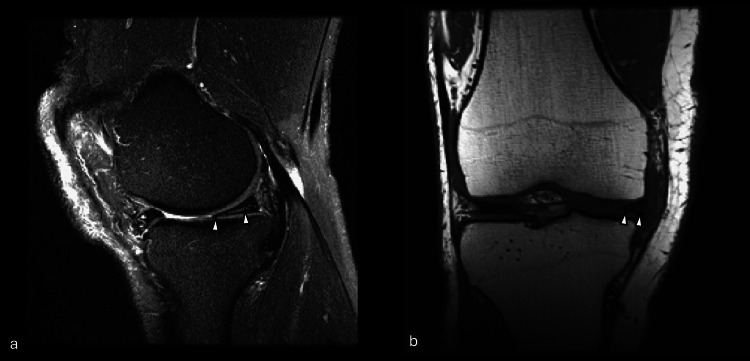
Example of MRI in which GPT-5 correctly identified a meniscal tear (true positive). Sample images from (a) sagittal plane T2-weighted series and (b) coronal plane T1-weighted series showing a knee with a meniscal tear (arrowheads). This example illustrates a true-positive case, correctly classified by GPT-5 as positive for a tear. GPT-5: generative pre-trained transformer 5; MRI: magnetic resonance imaging

**Figure 3:**
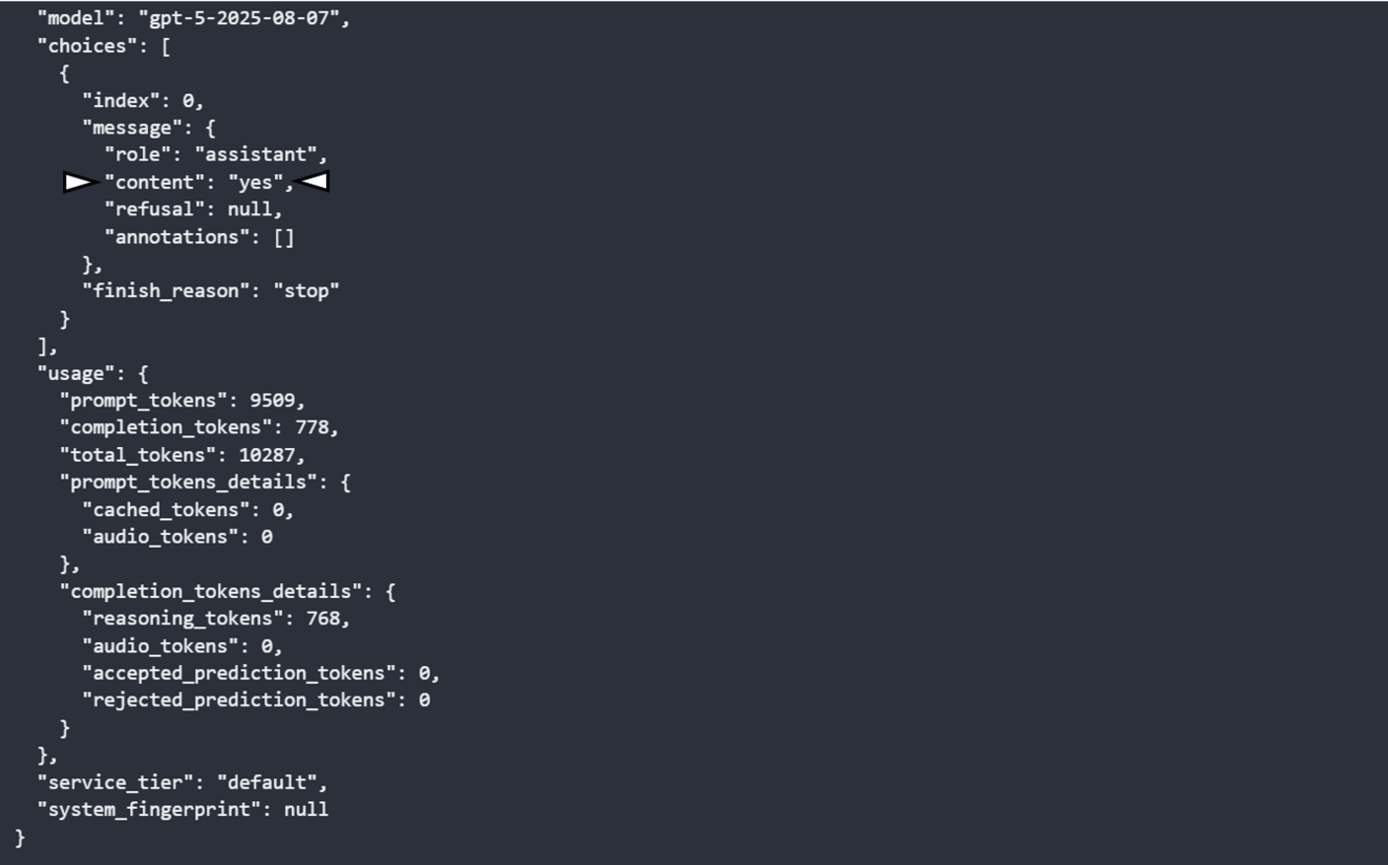
Example of OpenAI API response for the MRI slices input. This OpenAI API response showed that the gpt-5-2025-08-07 model successfully processed the prompt to analyze the input MRI slices and returned a simple reply ("yes") as shown at the "content" field (arrowhead). The detailed token usage is about 9,500 tokens for the input and 778 tokens for the completion, most of which were internal reasoning tokens. The request used the default service tier and encountered no errors or refusals. API: application programming interface; MRI: magnetic resonance imaging

Accuracy was 0.76 (95% CI: 0.668-0.833). Recall for tears (sensitivity) was 0.84 (95% CI: 0.715-0.917). Recall for intact meniscus (specificity) was 0.68 (95% CI: 0.542-0.792). The precision for the tear present (yes) class was 0.724 (95% CI: 0.598-0.822). The precision for the "no tear" prediction was 0.810 (95% CI: 0.667-0.900). The model's F1-score for detecting meniscal tears was 0.778 (95% CI: 0.681-0.859). For the "no tear" class, the F1-score was 0.739 (95% CI: 0.627-0.832). These metrics are summarized in Table 1. Examples of false-positive and true-positive cases are shown in Figure 1 and Figure 2. An example of a GPT response is shown in Figure 3.

**Table 1:** Performance of GPT-5 in detecting meniscal tears on knee MRI (n=100). Standard classification metrics including accuracy, precision, recall, F1-score, and support are shown. Each metric is shown with its 95% CI. CI: confidence interval; GPT-5: generative pre-trained transformer 5; MRI: magnetic resonance imaging

Class/metric	Precision (95% CI)	Recall (95% CI)	F1-score (95% CI)	Support (n)
Meniscal tear present	0.724 (0.598-0.822)	0.840 (0.715-0.917)	0.778 (0.681-0.859)	50
Meniscal tear absent	0.810 (0.667-0.900)	0.680 (0.542-0.792)	0.739 (0.627-0.832)	50
Overall accuracy	0.76 (0.668-0.833)	-	-	100

## Discussion

Overall diagnostic performance of GPT-5 on meniscal tear detection

In this study, GPT-5 demonstrated a moderate level of accuracy (76%) in detecting meniscal tears on knee MRI, approaching but not yet reaching the performance of specialized AI models or human experts. The result is inferior to CNN-based Stanford MRNet accuracy near 79-80%, which is trained specifically for MRI knee abnormality detection [3,6]. More recent deep learning models, including those employing graph convolutional networks (GCN) (KneeXNet) and attention mechanisms, have achieved AUC values exceeding 0.96 for meniscal tear detection on the MRNet dataset [7]. GPT-5's performance therefore falls short of these specialized models, which is expected given that GPT-5 is a general-purpose model applied in a zero-shot setting without domain-specific training, optimization, or fine-tuning.

With a recall rate of 84% for meniscal tears, GPT-5 identified a majority of tear cases. This performance falls within the lower range of reported radiologist sensitivities in the literature (often ~83-94% for meniscal tears, depending on the study) [2], noting differences in study populations and study designs. However, the specificity was substantially lower at 68%, which is below the typical radiologist performance (specificity ~82-93%) [2] and below some prior specialized deep learning models (e.g., MRNet model specificity ~74%) [3]. This imbalance in performance results in a relatively high false-positive rate.

Overview of GPT-5 and multimodal GPT architectures

GPT-5 is a proprietary large multimodal transformer model developed by OpenAI as part of the GPT family of generative pre-trained transformers. Like its predecessors, it is trained primarily on massive text corpora, but more recent GPT-4-series and GPT-5-series models also incorporate a vision encoder, enabling the model to accept images as input and reason over them jointly with text [8,9]. Although the exact architecture and training data for GPT-5 have not been publicly released, available technical reports on GPT-4 and GPT-4V describe a unified transformer backbone that processes both text tokens and image embeddings, allowing cross-modal attention and joint optimization on diverse image-text tasks [8,9].

Compared with earlier LLMs that were purely text-based, multimodal GPT models can directly interpret radiological images rather than relying on textual descriptions of imaging findings. Prior work with GPT-4V has shown that the model can answer visual questions, interpret charts, and attempt medical image analysis, but that its diagnostic performance in radiology remains limited [9,10].

Prior evaluations of LLMs for medical image interpretation

There is a rapidly growing body of literature evaluating LLMs for medical image interpretation. In one of the earlier musculoskeletal imaging assessments, Horiuchi et al. compared GPT-4-based ChatGPT (given textual imaging descriptions) with GPT-4V (given the images directly) and radiologists. GPT-4V achieved only 8% diagnostic accuracy across musculoskeletal MRI cases, whereas GPT-4 using textual findings performed substantially better, and expert radiologists achieved >90% accuracy, underscoring GPT-4V's inability to reliably interpret raw images [4].

Similarly, Ueda et al. examined GPT-4's diagnostic performance using patient history combined with textual imaging findings from the Radiology "Diagnosis Please" series. ChatGPT achieved 54% overall diagnostic accuracy, compared with the significantly higher performance of expert radiologists, who typically exceed 80-90% on the same cases [5].

More recent evaluations of state-of-the-art multimodal LLMs, including GPT-4o, Claude 3 Opus (Anthropic, San Francisco, CA, USA), and Gemini 1.5 Pro (Google DeepMind, London, UK), show incremental yet still limited improvements in radiologic image interpretation. In a 324 case analysis, Sonoda et al. reported primary diagnostic accuracies of 41% for GPT-4o, 54% for Claude 3 Opus, and 33.9% for Gemini 1.5 Pro, in contrast with radiologists assessing the same "Diagnosis Please" cases who typically achieve 75-90% accuracy. Even when broader differential diagnoses were considered (top-3 accuracy), LLM performance reached only 49-62%, remaining well below expert-level expectations [11]. Previous LLMs also struggled with foundational image-based tasks such as radiologic anatomy identification: Sarangi et al. evaluated ChatGPT-4 on 100 Fellowship of the Royal College of Radiologists (FRCR) Part 1-style radiological anatomy questions and found extremely low exact correctness (4% without context; 7.5% with context), despite perfect modality identification [12].

Moreover, even in studies where multimodal LLMs are assisted by structured imaging descriptions, supplemental clinical context, or multiple-choice answer formats, their diagnostic accuracy remains highly variable and consistently below that of human readers. Kim et al. report that current LLMs exhibit wide performance variability across radiologic tasks [13], while evaluations on Japanese diagnostic radiology board examinations demonstrate that multimodal models typically achieve only modest accuracy (often ~30-50%) compared with substantially higher radiologist performance [14].

Several systematic reviews have synthesized these findings, noting that early applications of ChatGPT and related LLMs in radiology show abilities for tasks such as report drafting, explanation of imaging findings, decision support, and medical education, but they also highlight persistent limitations, including factual errors, hallucinations, and inconsistent reasoning when confronted with complex imaging data [11,15,16].

Specialized deep learning systems for knee MRI pathology

Radiology-specific deep learning systems such as MRNet and KneeXNet use supervised training on task-specific labeled datasets and operate on multi-slice MRI. MRNet applies 2D CNNs slice-wise to each MRI series (sagittal T2, coronal T1, axial proton density), aggregates slice features, and uses a logistic-regression meta-classifier to predict overall abnormality, ACL tear, and meniscal tear. It achieved AUCs of 0.937 for abnormality, 0.965 for ACL tears, and 0.847 for meniscal tears and provided modest improvements in clinician performance when used as an assistive tool [3].

More recent systems such as KneeXNet extend this approach by representing the knee joint as a graph of anatomical landmarks and applying graph convolution layers with a multi-scale feature fusion model to capture spatial dependencies across slices and imaging planes. Trained on the MRNet dataset, KneeXNet achieves AUCs >0.96 for meniscal tear detection and can generate class activation maps to localize suspected tears [7]. Other multi-task deep learning systems have been developed to classify multiple knee abnormalities on MRI (e.g., cartilage defects, bone marrow edema, ligament injuries) within a single network [17].

These dedicated radiology models differ fundamentally from GPT-5. First, they are trained end-to-end on thousands of knee MRI examinations labeled specifically for structural knee injuries [3,7,17]. Second, their architecture was explicitly designed for 3D medical imaging, with slice-order modeling, multi-sequence fusion, and attention mechanisms tailored to volumetric data [3,7,17]. Third, they are evaluated with receiver operating characteristic (ROC) curve analysis and calibrated operating points that allow clinicians to choose sensitivity and specificity thresholds appropriate for clinical use. Finally, many of these models provide visual explanations (e.g., class activation maps) that highlight regions contributing most to the prediction, improving transparency and trust [3,7]. GPT-5, in contrast, is a general-purpose multimodal LLM without knee-specific training, explicit volumetric modeling, or built-in visual explainability.

Black-box limitations and lack of visual explainability

A key concern is the black-box nature of GPT-5's decisions. Unlike MRNet, KneeXNet, and related CNN/GCN-based models, which can generate class activation maps or heatmaps that localize suspected tears, GPT-5 currently provides only a global textual decision without indicating where the tear is located or which slices are most influential [3,7]. This lack of visual interpretability limits its immediate clinical usability and makes it difficult to understand failure modes such as whether the model confuses artifacts (e.g., magic-angle artifacts), vascular structures, or postoperative changes with true tears.

Future work should examine integrating GPT-5 with explainable vision modules or hybrid pipelines such as a radiology-specific CNN which generates lesion maps that the LLM then interprets.

Clinical implications of GPT-5's performance on meniscal tear detection

Despite these limitations, GPT-5's performance has several potential clinical implications, particularly within human-in-the-loop workflows. Its relatively high sensitivity (84%) indicates that, with further validation and calibration, GPT-5 could function as a triage aid or second-reader system. In this role, the model might pre-screen knee MRI studies and flag examinations with a higher likelihood of meniscal tear for earlier human review. However, the model's low specificity and corresponding 32% false-positive rate in a balanced dataset raise important concerns. In routine clinical settings, where the prevalence of meniscal tears is typically lower, this would likely exacerbate false alarms and potentially reduce reading efficiency.

From a workflow perspective, GPT-5 may offer value as decision support, especially in low-resource environments where musculoskeletal imaging expertise is limited. A tool capable of reliably highlighting potentially abnormal cases could assist general radiologists or orthopedic clinicians, provided that its output is interpreted cautiously and under appropriate human oversight. This aligns with prior literature showing that LLMs such as ChatGPT can support tasks, including case analysis, educational explanation of imaging findings, and draft report generation, yet are not appropriate for autonomous diagnostic use [15,18,19].

Although AI tools can theoretically reduce reading time by highlighting abnormal cases, GPT-5's low specificity means many normal studies may be incorrectly flagged, potentially increasing the number of cases requiring re-checks and possibly prolonging interpretation time. This risk of unnecessary follow-up and possible overdiagnosis underscores that GPT-5 is best suited as a supportive tool, used within a supervised, human-in-the-loop workflow rather than as a stand-alone diagnostic system.

Regulatory and ethical considerations

The clinical adoption of GPT-5 also involves regulatory and ethical considerations. General-purpose LLMs do not align neatly with existing Food and Drug Administration (FDA)-style medical AI validation frameworks, which require task-specific training, fixed model behavior, and transparent performance metrics [20]. The limited disclosure of training data and the dynamic updating typical of commercial LLMs complicate reproducibility and risk assessment [21]. Moreover, potential biases arising from non-medical pre-training corpora may affect generalizability across scanners, clinical settings, and patient populations, reinforcing known disparities in medical AI systems [22]. These issues underscore the need for rigorous external validation, post-deployment monitoring, and maintained human oversight before models like GPT-5 can be safely integrated into clinical workflows.

Study limitations

Our study has several limitations. First, the sample size is relatively small (100 cases), which makes performance estimates more susceptible to random variation, limits statistical power, may reduce the robustness and generalizability of the findings, and may not capture the full spectrum of meniscal tear appearances, mimics (e.g., meniscocapsular separation, meniscal contusions), or postoperative changes.

Second, we used only two MRI planes, including axial images or additional sequences (e.g., 3D isotropic sequences) that might improve diagnostic performance. Third, we used a strict binary yes/no question prompt, which did not allow GPT-5 to express uncertainty. In clinical practice, equivocal findings are common, and AI systems may benefit from probabilistic or multi-category outputs. Fourth, unlike real clinical practice, GPT-5 was not provided with patient history, age, mechanism of injury, or clinical symptoms. These contextual factors influence radiologic interpretation and can improve diagnostic accuracy [2]. Fifth, the dataset in this study was deliberately balanced by class (50 tears and 50 non-tears). This does not reflect the true prevalence of meniscal tears in routine clinical practice and may bias estimates of performance. Also, GPT-5 is a proprietary, cloud-hosted model whose inner workings are not fully transparent and may change over time. Any clinical use would require continuous monitoring, robust external validation across diverse sites, and careful attention to ethical and medico-legal considerations [15,18].

Also, as a pilot study, the work lacks full clinical realism, as the evaluation isolated meniscal tears as a single binary classification task without accounting for other common co-existing knee pathologies such as ACL tears, cartilage lesions, bone marrow edema, or joint effusions. Real clinical cases rarely present with only one abnormality, and the simplified task may overestimate model performance. Furthermore, the study did not include subgroup or case-level analyses, such as stratifying performance by tear pattern (e.g., radial, complex, bucket-handle) or compartmental distribution, limiting insight into how GPT-5 performs across the heterogeneous presentations encountered in routine musculoskeletal imaging.

A further limitation concerns the ground-truth labels. Although the MRNet validation set uses majority-vote labels from three senior musculoskeletal radiologists who reviewed all DICOM series, clinical reports, and follow-up imagings, these labels were not arthroscopically confirmed. Consequently, misclassification relative to the surgical gold standard is still possible, which may influence the apparent diagnostic performance of GPT-5.

Additionally, we did not compare GPT-5's performance with that of expert radiologists on the same cases, limiting our ability to contextualize its accuracy against human benchmarks.

Finally, a further limitation is the lack of transparency regarding GPT-5's training data. As with other proprietary LLMs, OpenAI does not disclose the datasets used for pre-training or multimodal vision tuning [8], so it is unknown whether GPT-5 was exposed to portions of the MRNet dataset. Although general-purpose multimodal models are usually trained on broad, heterogeneous image-text corpora rather than specialized medical imaging data [23], some degree of dataset overlap cannot be excluded, which introduces uncertainty about potential data leakage and may artificially inflate the model's apparent performance.

## Conclusions

In this pilot study, although GPT-5 exhibits potential in knee MRI meniscal tear analysis, achieving an F1-score of approximately 0.78 and an overall accuracy of 76%, the results should be interpreted cautiously due to study limitations, and its current clinical utility remains limited. Although these metrics represent a significant advancement over previous multimodal LLMs, the model's high false-positive rate and resultant low specificity preclude its use as an autonomous diagnostic tool. Nevertheless, this pilot evaluation provides an initial proof of concept that general-purpose multimodal LLMs may possess emerging capabilities for direct MRI interpretation. With further refinement to reduce false alarms and improved explainability, successor LLMs could become valuable diagnostic assistants in musculoskeletal radiology.
